# Changes in bone marrow fibrosis during momelotinib or ruxolitinib therapy do not correlate with efficacy outcomes in patients with myelofibrosis

**DOI:** 10.1002/jha2.854

**Published:** 2024-02-05

**Authors:** Stephen T. Oh, Srdan Verstovsek, Vikas Gupta, Uwe Platzbecker, Timothy Devos, Jean‐Jacques Kiladjian, Donal P. McLornan, Andrew Perkins, Maria Laura Fox, Mary Frances McMullin, Adam J. Mead, Miklos Egyed, Jiri Mayer, Tomasz Sacha, Jun Kawashima, Mei Huang, Bryan Strouse, Ruben Mesa

**Affiliations:** ^1^ Division of Hematology Washington University School of Medicine St. Louis Missouri USA; ^2^ Department of Leukemia The University of Texas MD Anderson Cancer Center Houston Texas USA; ^3^ Department of Medicine, Princess Margaret Cancer Centre University of Toronto Toronto Canada; ^4^ Clinic of Hematology, Cellular Therapy, and Hemostaseology University of Leipzig Medical Center Leipzig Germany; ^5^ Microbiology, and Immunology, Laboratory of Molecular Immunology (Rega Institute) Department of Hematology, University Hospitals Leuven and Department of Microbiology and Immunology, Laboratory of Molecular Immunology (Rega Institute), KU Leuven Leuven Belgium; ^6^ Université Paris Cité, AP‐HP, Hôpital Saint‐Louis, Centre d’Investigations Cliniques Paris France; ^7^ Department of Haematology Guy's and St Thomas’ NHS Foundation Trust and University College Hospital London UK; ^8^ Australian Centre for Blood Diseases Monash University Melbourne Australia; ^9^ Department of Haematology Vall d'Hebron University Hospital Barcelona Spain; ^10^ Centre for Medical Education Queen's University Belfast UK; ^11^ MRC Molecular Haematology Unit MRC Weatherall Institute of Molecular Medicine NIHR Biomedical Research Centre University of Oxford Oxford UK; ^12^ Department of Hematology Somogy County Kaposi Mór General Hospital Kaposvár Hungary; ^13^ Department of Internal Medicine, Hematology and Oncology Masaryk University and University Hospital Brno Brno Czech Republic; ^14^ Department of Hematology Jagiellonian University Hospital Kraków Poland; ^15^ Sierra Oncology, a GSK company San Mateo California USA; ^16^ Atrium Health Wake Forest Baptist Comprehensive Cancer Center Wake Forest University School of Medicine Winston‐Salem North Carolina USA

**Keywords:** bone marrow fibrosis, JAK inhibitor, momelotinib, myelofibrosis, ruxolitinib

## Abstract

Bone marrow fibrosis (BMF) is a pathological feature of myelofibrosis, with higher grades associated with poor prognosis. Limited data exist on the association between outcomes and BMF changes. We present BMF data from Janus kinase (JAK) inhibitor–naive patients from SIMPLIFY‐1 (NCT01969838), a double‐blind, randomized, phase 3 study of momelotinib vs ruxolitinib. Baseline and week 24 bone marrow biopsies were graded from 0 to 3 as per World Health Organization criteria. Other assessments included Total Symptom Score, spleen volume, transfusion independence status, and hemoglobin levels. Paired samples were available from 144 and 160 patients randomized to momelotinib and ruxolitinib. With momelotinib and ruxolitinib, transfusion independence was achieved by 87% and 44% of patients with BMF improvement of ≥1 grade and 76% and 56% of those with stable/worsening BMF; there was no association between BMF changes and transfusion independence for either arm (momelotinib, *p* = .350; ruxolitinib, *p* = .096). Regardless of BMF changes, hemoglobin levels also generally increased on momelotinib but decreased on ruxolitinib. In addition, no associations between BMF changes and spleen (momelotinib, *p* = .126; ruxolitinib, *p* = .407)/symptom (momelotinib, *p* = .617; ruxolitinib, *p* = .833) outcomes were noted, and no improvement in overall survival was observed with ≥1‐grade BMF improvement (momelotinib, *p* = .395; ruxolitinib, *p* = .407). These data suggest that the anemia benefit of momelotinib is not linked to BMF changes, and question the use of BMF assessment as a surrogate marker for clinical benefit with JAK inhibitors.

## 1 INTRODUCTION

Myelofibrosis is a rare Philadelphia chromosome–negative myeloproliferative neoplasm that may present de novo or secondary to polycythemia vera or essential thrombocythemia [1, 2]. Myelofibrosis is associated with poor prognosis and presents with heterogenous clinical manifestations, including anemia, thrombocytopenia, splenomegaly, bone marrow fibrosis (BMF), and debilitating constitutional symptoms (e.g., fatigue, fevers, and night sweats) [3, 4, 5, 6]. Up to 90% of patients with myelofibrosis harbor somatic mutations in the driver genes *JAK2*, *CALR*, or *MPL*, resulting in constitutive activation of the Janus kinase (JAK)‐signal transducer and activator of transcription (STAT) signaling pathway, most commonly gain‐of‐function *JAK2* V617F mutation [3, 4, 7]. Dysregulated JAK‐STAT signaling drives the production of inflammatory cytokines and clonal proliferation, leading to extramedullary hematopoiesis and splenomegaly [2, 8, 9]. Leukemic transformation is a potential complication of myelofibrosis and is almost always fatal, with 20% of primary myelofibrosis cases evolving into acute myeloid leukemia [5, 10, 11].

JAK inhibitors have been the mainstay of myelofibrosis treatment for over a decade. Ruxolitinib was the first JAK1/2 inhibitor to receive US Food and Drug Administration approval in 2011 [12]. Fedratinib, an inhibitor of JAK2 and FMS‐like tyrosine kinase 3 (FLT3), and pacritinib, which inhibits JAK2, FLT3, interleukin 1 receptor‐associated kinase 1 (IRAK1), and activin A receptor type 1 (ACVR1) [3, 13, 14], were subsequently approved in 2019 and 2022, respectively [15, 16]. These JAK inhibitors have demonstrated spleen size reduction and symptom improvement in patients with myelofibrosis, particularly when high dose intensity can be maintained [17, 18, 19, 20]. However, some have also been associated with potentially dose‐limiting myelosuppression, worsening anemia, and decreasing platelet counts, especially in patients who may already have anemia and/or thrombocytopenia [12, 15, 16, 21–24]. Momelotinib, an inhibitor of JAK1, JAK2, and ACVR1, was approved in 2023 for the treatment of patients with myelofibrosis and anemia [25] and has shown spleen and symptom benefits in phase 3 trials in this patient population [26, 27, 28].

BMF is a key histopathological feature and major diagnostic criterion of myelofibrosis and is characterized by increased deposition of reticulin and/or collagen fibers secondary to aberrant immature megakaryocytes, which release excessive amounts of inflammatory and fibrogenic cytokines [3, 6, 29–31]. Bone marrow biopsies are evaluated and scored using updated World Health Organization criteria consisting of 4 escalating grades of severity: grade 0–3 [32]. BMF grading, while not incorporated in conventional prognostic score systems (International Prognostic Scoring System [IPSS] or Dynamic International Prognostic Scoring System [DIPSS]), does form part of the more recent Mutation‐Enhanced International Prognostic Scoring System 70 and 70‐plus (MIPPS70 and MIPPS70+) [3, 33]. Clinical studies have associated higher grades of BMF with poor prognosis [29, 34, 35]. Recent studies have reported BMF improvement as evidence of disease modification, and BMF change or reversal has been pursued as an endpoint in clinical trials, including after allogeneic transplantation [4, 36–39]. However, to date, clinical data on associations of treatment‐related BMF changes with efficacy outcomes (e.g., survival and symptom, spleen, and anemia responses) are limited, and the role of BMF in the setting of JAK inhibitor therapy is not clear.

This study investigates the impact of two differentiated JAK inhibitors, momelotinib and ruxolitinib, on BMF grade and assesses if any correlations exist between BMF grade changes and clinical outcomes among JAK inhibitor–naive patients with myelofibrosis in the phase 3 SIMPLIFY‐1 trial (NCT01969838).

## 2 MATERIALS AND METHODS

### 2.1 Patients

The study design of SIMPLIFY‐1, a randomized, head‐to‐head, double‐blind, phase 3 study of momelotinib vs ruxolitinib, has been described elsewhere [27]. In brief, 432 JAK inhibitor–naive patients with primary myelofibrosis, post–polycythemia vera myelofibrosis, or post–essential thrombocythemia myelofibrosis were randomized 1:1 to receive momelotinib or ruxolitinib (Figure S1) [27]. This JAK inhibitor–naive setting was utilized in the present analysis to minimize confounders from prior treatments. The study was approved by the institutional review boards and independent ethics committees at each study site, and all participants provided written informed consent. Additional signatures were collected for the use of biopsies in other biomarker analyses.

### 2.2 Bone marrow aspirate and biopsy

Bone marrow aspirate and biopsies were performed prior to treatment initiation (baseline), after 24 weeks of randomized treatment (within a window of ± 7 days) with either momelotinib or ruxolitinib, at week 96, and as required to assess response per International Working Group‐Myeloproliferative Neoplasms Research and Treatment and European LeukemiaNet criteria [40]. Patients continued treatment until the completion of this visit. Bone marrow aspirate and biopsy samples were assessed by a local hematopathologist for BMF grading using the revised 2016 World Health Organization classification and diagnostic criteria for myeloproliferative neoplasms; assessment included reticulin (e.g., silver stain) and collagen fibers (e.g., trichrome stain) from grade 0 (normal bone marrow) to grade 3 (diffuse and dense increase in reticulin; coarse bundles of thick fibers consistent with collagen) [32].

### 2.3 Additional analyses

As described previously [27], the rate of ≥35% spleen volume reduction (assessed by magnetic resonance imaging or computed tomography and evaluated by a blinded central reader), the rate of ≥50% reduction in Total Symptom Score (TSS), the rate of transfusion independence, and hemoglobin levels were assessed. For this analysis, baseline and week 24 data were used.

### 2.4 Statistical analysis

χ^2^ test was used to assess the association between BMF change and both transfusion independence response and spleen/symptom response at week 24 in each arm. To examine whether BMF change at week 24 was predictive of overall survival (OS), OS was calculated from week 24, analyzed using the Kaplan‐Meier analyses, and compared between groups by BMF change at week 24 with log‐rank tests and proportional hazard Cox regression models. All analyses were descriptive.

## 3 RESULTS

### 3.1 BMF grades at baseline

**FIGURE 1:**
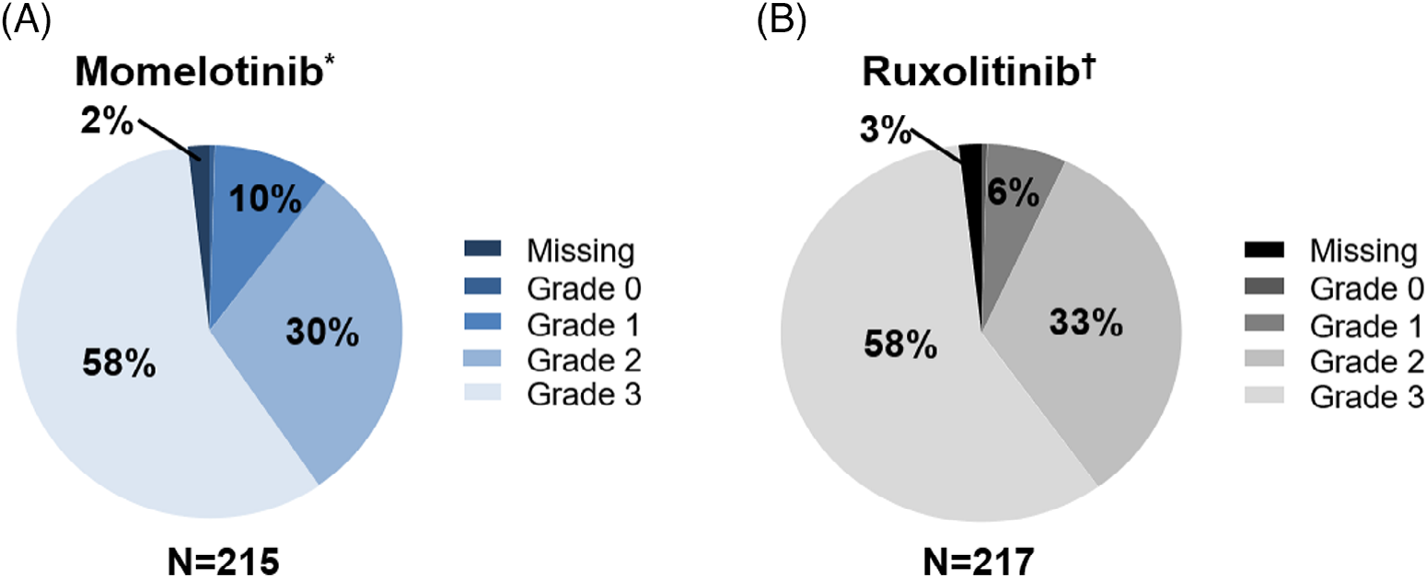
BMF grades at baseline in JAK inhibitor–naive patients (*N* = 432) in SIMPLIFY‐1. (A) Randomized to momelotinib. (B) Randomized to ruxolitinib. ^*^A total of 211/215 momelotinib‐randomized patients had baseline BMF assessment; 1 patient had grade 0 BMF. ^†^A total of 213/217 ruxolitinib‐randomized patients had baseline BMF assessment; 1 patient had grade 0 BMF. BMF, bone marrow fibrosis; JAK, Janus kinase.

Of the 432 JAK inhibitor–naive patients in SIMPLIFY‐1 randomized to momelotinib (*N* = 215) or ruxolitinib (*N* = 217), 98% had baseline BMF assessments (momelotinib, *n* = 211; ruxolitinib, *n* = 213) (Figure 1). In each arm, 58% of randomized patients had grade 3 BMF (momelotinib, *n* = 124; ruxolitinib, *n* = 126). Only 1 patient in each arm had grade 0 BMF. The median time since diagnosis of myelofibrosis was 1.5 years (interquartile range, 0.4–3.9 years).

**FIGURE 2:**
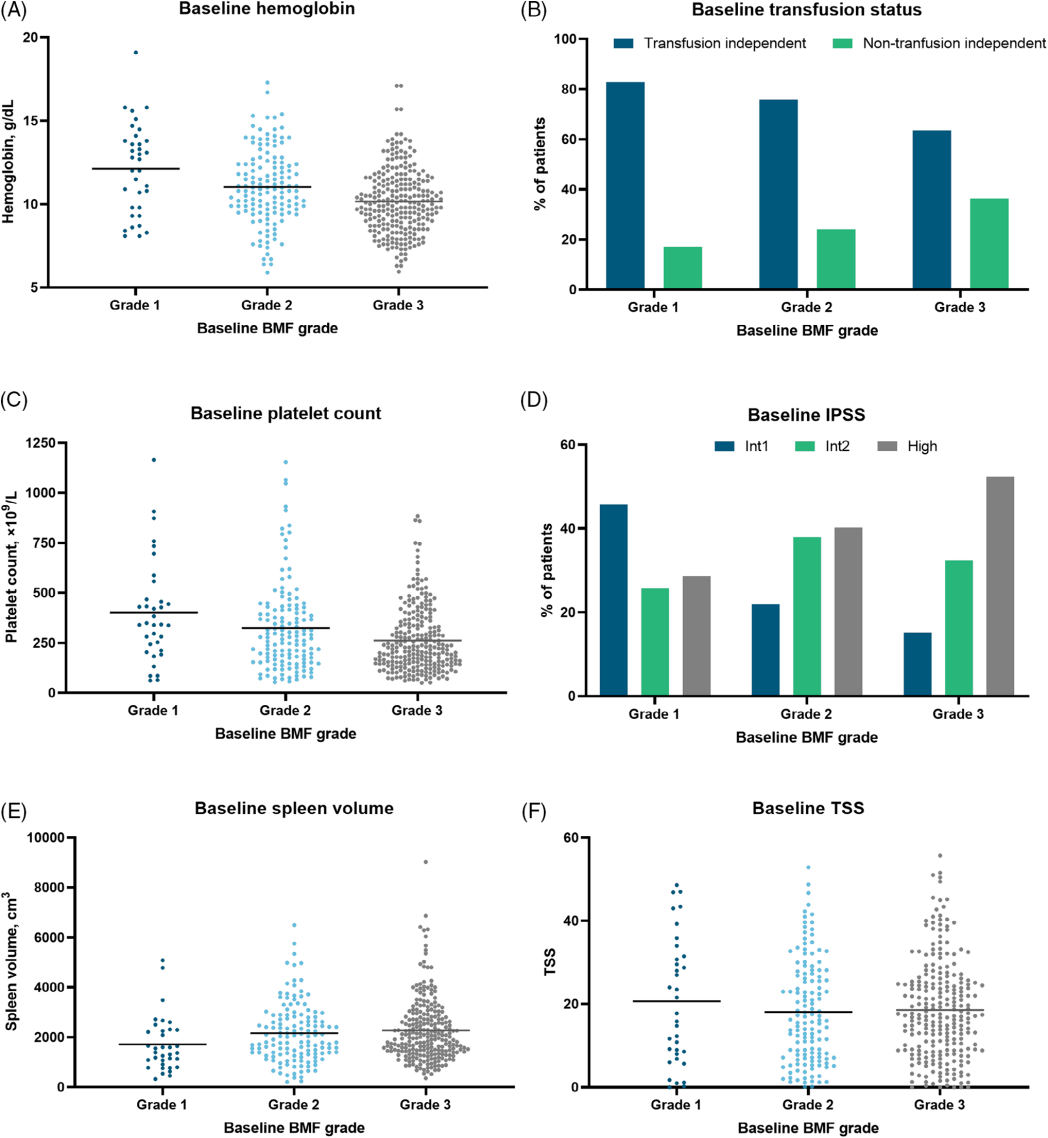
Overall SIMPLIFY‐1 population by BMF grade at baseline. (A) Hemoglobin. (B) Transfusion status. (C) Platelet counts. (D) IPSS. (E) Spleen volume. (F) TSS. BMF, bone marrow fibrosis; Int1, intermediate 1; Int2, intermediate 2; IPSS, International Prognostic Scoring System; TSS, Total Symptom Score.

Hemoglobin level, transfusion status, platelet count, IPSS score, spleen volume, and TSS at baseline by BMF grade at baseline for both arms are shown in Figure 2. Higher baseline BMF grading trended toward lower hemoglobin levels (Figure 2A), rates of transfusion independence (Figure 2B), and platelet counts (Figure 2C) at baseline. IPSS score and BMF grade trended similarly with regard to severity (Figure 2D); a greater proportion of patients with grade 1 BMF had intermediate‐1–risk myelofibrosis, whereas a greater proportion of patients with grade 3 BMF had high‐risk myelofibrosis. Higher BMF grading trended toward greater spleen volume (Figure 2E) but not higher TSS (Figure 2F).

### 3.2 BMF grade changes from baseline to week 24

**FIGURE 3:**
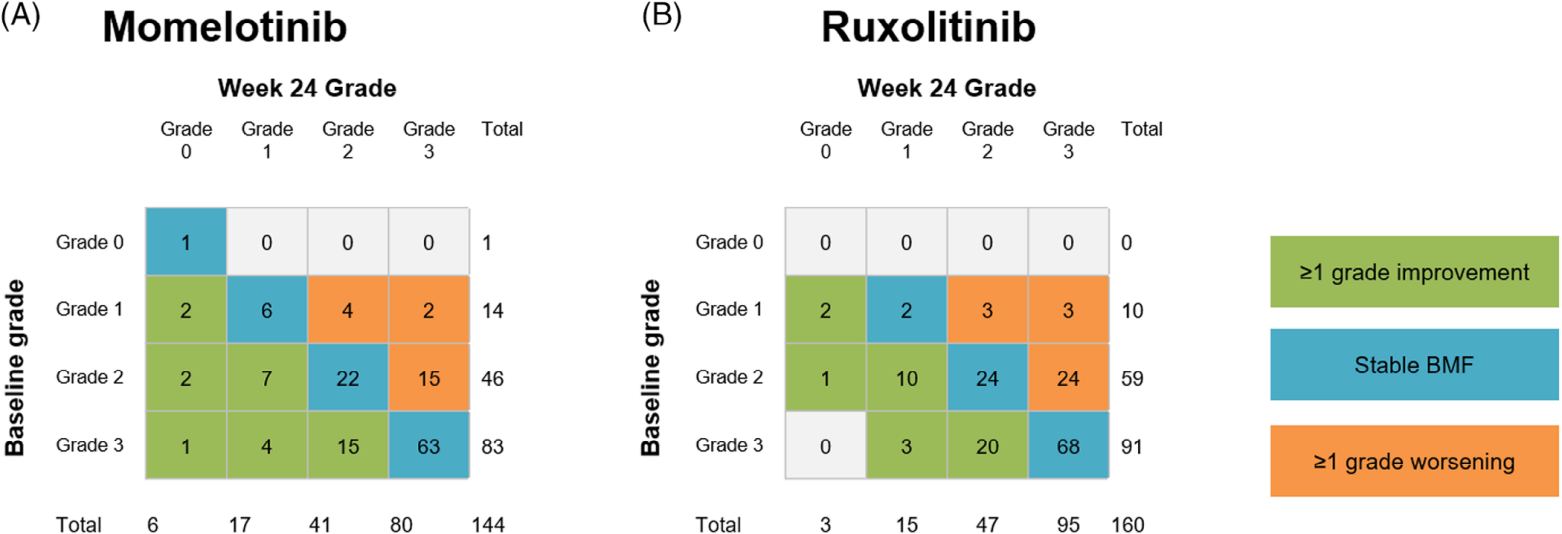
Change in BMF grade from baseline to week 24 in JAK inhibitor–naive patients in SIMPLIFY‐1. (A) Patients treated with momelotinib. (B) Patients treated with ruxolitinib. BMF, bone marrow fibrosis; JAK, Janus kinase.

In SIMPLIFY‐1, 144 of 215 (67%) and 160 of 217 (74%) patients randomized to momelotinib and ruxolitinib, respectively, had a paired biopsy at week 24 for analysis. Of the 144 patients in the momelotinib arm, 31 (22%) had a ≥1‐grade improvement in BMF (Figure 3A); of the 160 patients in the ruxolitinib arm, 36 (23%) had a ≥1‐grade improvement in BMF (Figure 3B). Momelotinib and ruxolitinib had similar effects in maintaining/improving BMF, with 85% (123/144) and 81% (130/160) of patients, respectively, having stable or improved BMF in each arm. BMF grading was also available for 43 additional paired samples from the momelotinib‐randomized arm at open‐label week 96. There was limited change to the overall BMF grades at week 24 (11 improving and nine worsening).

### 3.3 BMF grade changes and symptom and spleen response at week 24

**FIGURE 4:**
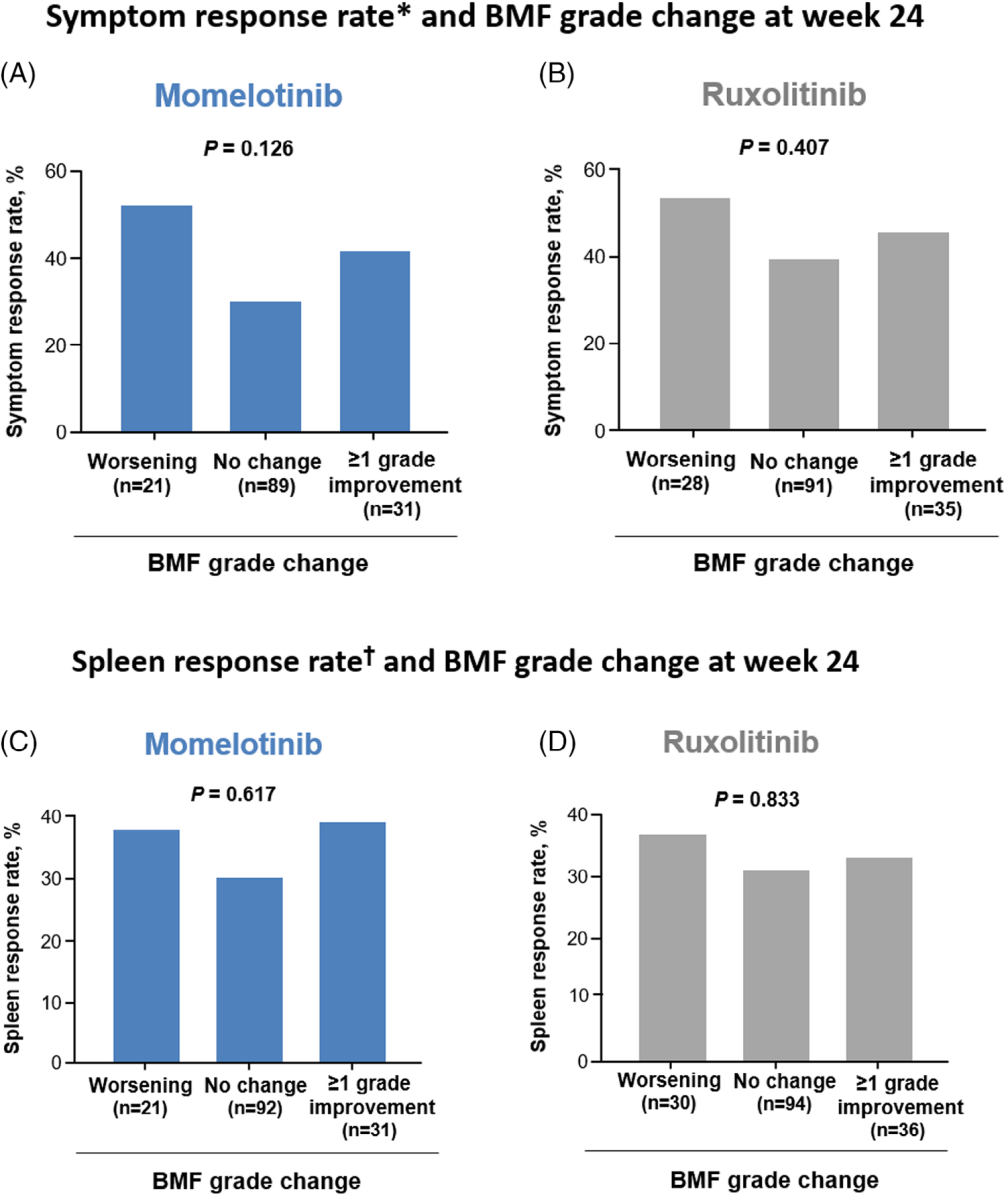
Proportion of JAK inhibitor–naive patients in SIMPLIFY‐1 who achieved symptom and spleen response by change in BMF grade from baseline to week 24. (A, C) Patients treated with momelotinib. (B, D) Patients treated with ruxolitinib. *Symptom response is defined as achieving a ≥50% reduction in TSS over the 28 days immediately before the end of week 24 compared with baseline. The percentage is calculated using the BMF change category as the denominator (i.e., ≥1‐grade improvement, no change, or worsening). ^†^Spleen response is defined as achieving a ≥35% reduction in spleen volume from baseline. The percentage is calculated using the BMF change category as the denominator (i.e., ≥1‐grade improvement, no change, or worsening). The *p*‐value was calculated using a *χ* ^2^‐test. BMF, bone marrow fibrosis; JAK, Janus kinase; TSS, Total Symptom Score.

No clear associations between BMF grade changes from baseline to week 24 and symptom response, defined as a ≥50% reduction in TSS at week 24, were observed in either treatment arm (Figure 4A,B). In the momelotinib arm (*n* = 141; Figure 4A), of the 31 patients (22%) with a ≥1‐grade improvement in BMF, 13 (42%) achieved a symptom response; of the 89 patients (63%) with stable BMF, 21 (30%) achieved a symptom response; and of the 27 patients (15%) with worsening BMF, 11 (52%) had a symptom response (*p* = .1257). In the ruxolitinib arm (*n* = 154; Figure 4B), of the 35 patients (23%) with a ≥1‐grade improvement in BMF, 16 (46%) achieved a symptom response; of the 91 patients (59%) with stable BMF, 36 (40%) achieved a symptom response; and of the 28 patients (18%) with worsening BMF, 15 (54%) had a symptom response (*p* = .4066).

Similarly, no clear associations between BMF grade changes from baseline to week 24 and spleen response, defined as a reduction of ≥35% in spleen volume at week 24, were observed in either treatment arm (Figure 4C,D). In the momelotinib arm (*n* = 144; Figure 4C), of the 31 patients (22%) with a ≥1‐grade improvement in BMF, 12 (39%) achieved a spleen response; of the 92 (64%) with stable BMF, 28 (30%) achieved a spleen response; and of the 21 (15%) with worsening BMF, eight (38%) achieved a spleen response (*p* = .6171). In the ruxolitinib arm (*n* = 160; Figure 4D), of the 36 patients (23%) with a ≥1‐grade improvement in BMF, 12 (33%) achieved a spleen response; of the 94 (59%) with stable BMF, 29 (31%) achieved a spleen response; and of the 30 (19%) with worsening BMF, 11 (37%) achieved a spleen response (*p* = .8331).

### 3.4 BMF grade changes and transfusion independence at week 24

**FIGURE 5:**
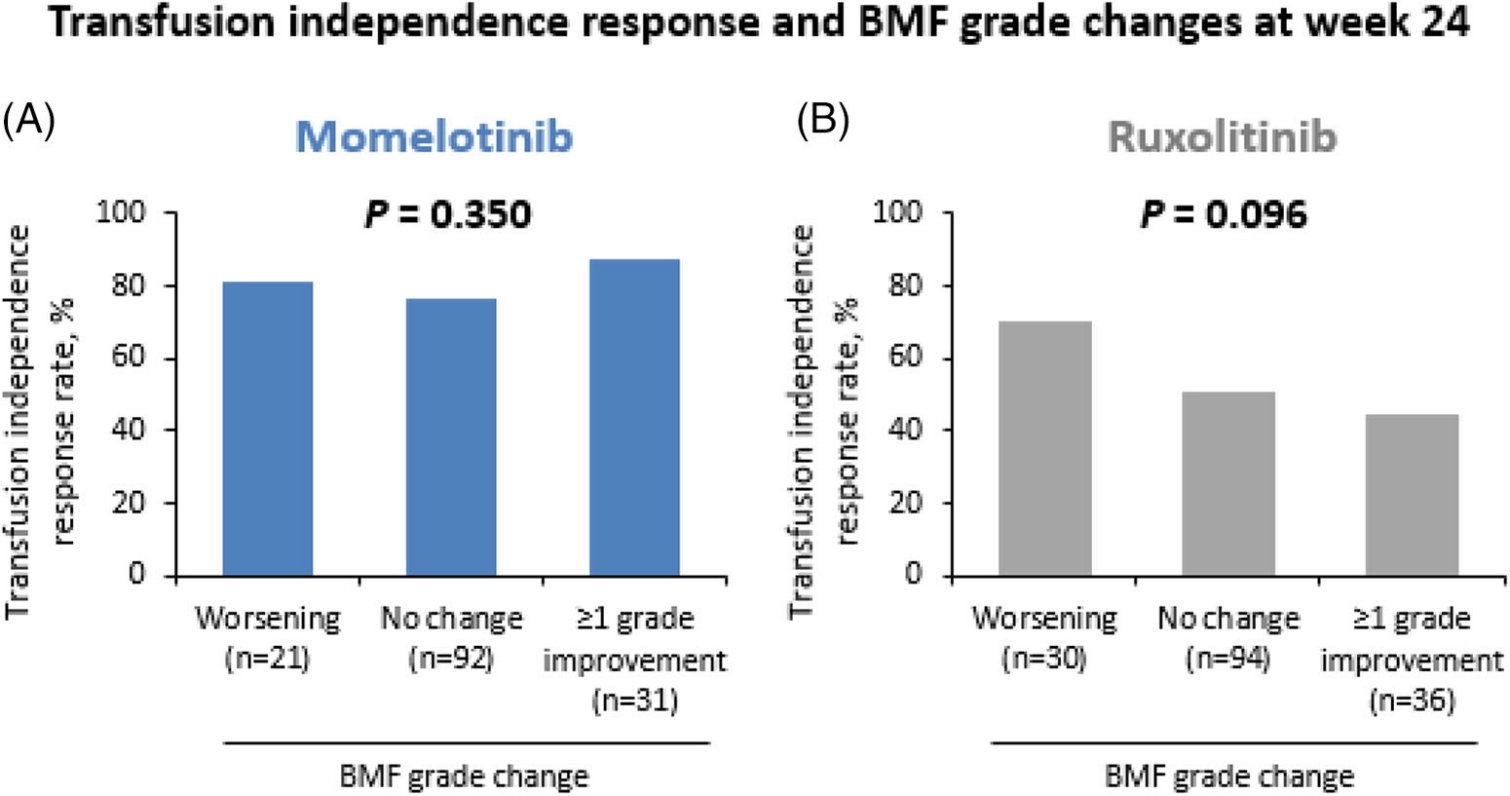
Proportion of JAK inhibitor–naive patients in SIMPLIFY‐1 who achieved transfusion independence response by change in BMF grade from baseline to week 24. (A) Patients treated with momelotinib. (B) Patients treated with ruxolitinib. Transfusion independence response was defined as the absence of red blood cell transfusions and no hemoglobin levels < 8 g/dL in the 12 weeks before week 24. The *p*‐value was calculated using a *χ* ^2^‐test. BMF, bone marrow fibrosis; JAK, Janus kinase.

Transfusion independence, defined as the absence of transfusions and no hemoglobin levels < 8 g/dL in the 12 weeks before week 24, was achieved by 78% of patients (113/144) in the momelotinib arm vs 53% (85/160) in the ruxolitinib arm in the analysis population with paired BMF grade at baseline and week 24. Of the 31 patients in the momelotinib arm with a ≥1‐grade improvement in BMF, 27 (87%) achieved transfusion independence at week 24 (Figure 5A); of the 92 patients with stable BMF, 69 (75%) achieved transfusion independence at week 24; of the 21 patients with worsening BMF, 17 (81%) achieved transfusion independence at week 24. In the ruxolitinib arm, of the 36 patients with a ≥1‐grade improvement in BMF, 16 (44%) achieved transfusion independence at week 24 (Figure 5B); of the 94 patients with stable BMF, 48 (51%) achieved transfusion independence at week 24; and of the 30 patients with worsening BMF, 21 (70%) achieved transfusion independence at week 24. Nearly twice as many patients on momelotinib vs ruxolitinib with a ≥1‐grade BMF improvement achieved transfusion independence at week 24 (87% [27/31] vs 44% [16/36]). For those with worsening BMF, the transfusion independence rate was higher in the momelotinib arm compared with the ruxolitinib arm (81% [17/21] vs 70% [21/30]). In patients treated with momelotinib, transfusion independence was achieved regardless of the extent of changes in BMF.

### 3.5 BMF grade changes and hemoglobin at week 24

**FIGURE 6:**
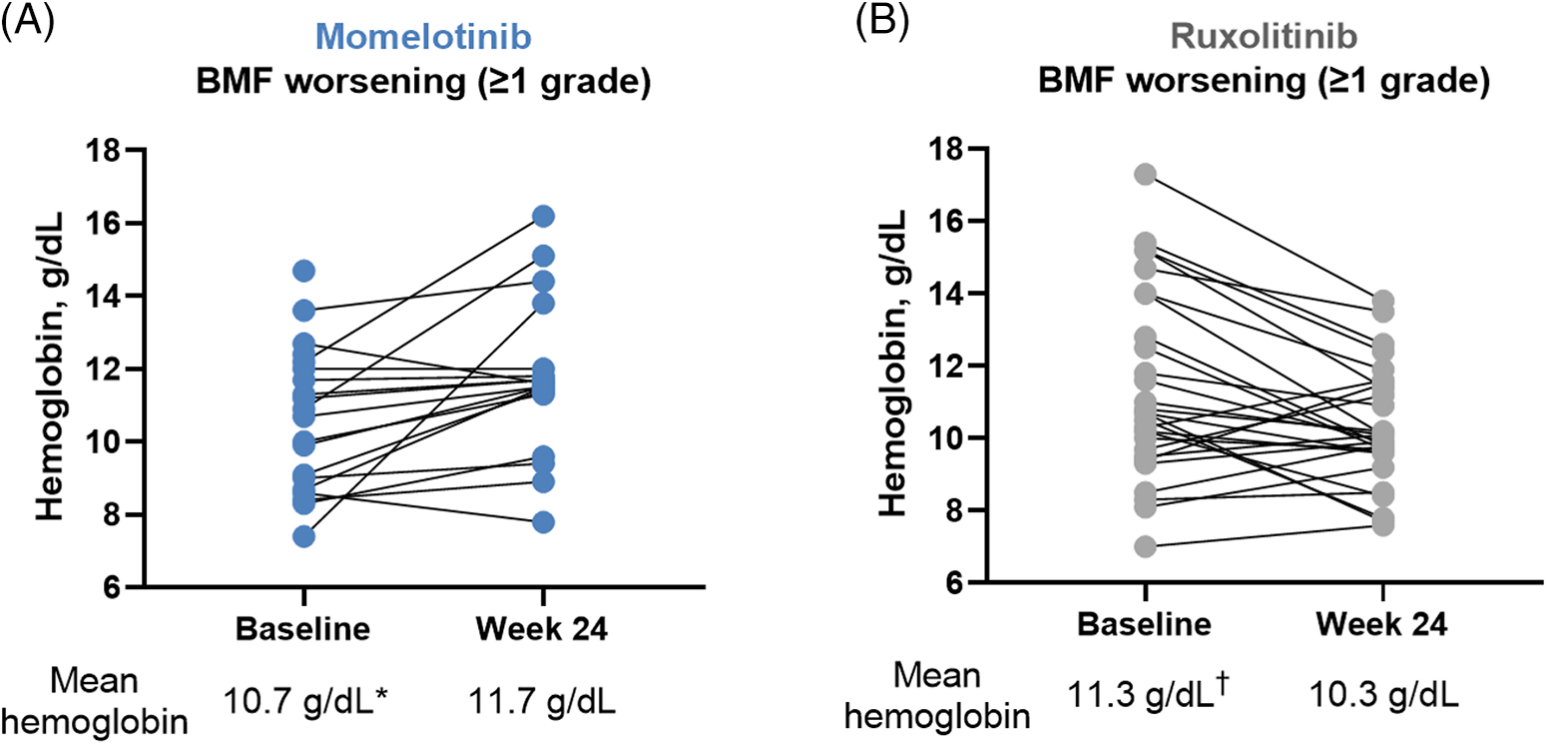
Hemoglobin levels at baseline and week 24 in JAK inhibitor–naive patients in SIMPLIFY‐1 with worsening BMF grade from baseline to week 24. (A) Patients treated with momelotinib. (B) Patients treated with ruxolitinib. *A total of 3/21 patients were missing week 24 hemoglobin measurement. ^†^A total of 2/30 patients were missing week 24 hemoglobin measurement. BMF, bone marrow fibrosis; JAK, Janus kinase.

Hemoglobin levels at baseline and week 24 by changes in BMF grade are shown in Figure 6 and Figure S2. In the momelotinib arm, hemoglobin levels increased regardless of the extent of changes in BMF; changes from baseline to week 24 were 10.7 to 11.7, 11.0 to 11.7, 11.3 to 12.4, and 9.5 to 10.5 (mean level in g/dL) with a ≥1‐grade worsening, 1‐grade improvement, 2‐grade improvement, and 3‐grade improvement, respectively. In the ruxolitinib arm, hemoglobin levels generally decreased with both improving and worsening BMF; changes from baseline to week 24 were 11.3 to 10.3, 10.6 to 9.6, and 11.5 to 9.9 (mean level in g/dL) with ≥1‐grade worsening, 1‐grade improvement, and 2‐grade improvement, respectively; there were no patients with a 3‐grade improvement in the ruxolitinib arm.

### 3.6 BMF and OS

**FIGURE 7:**
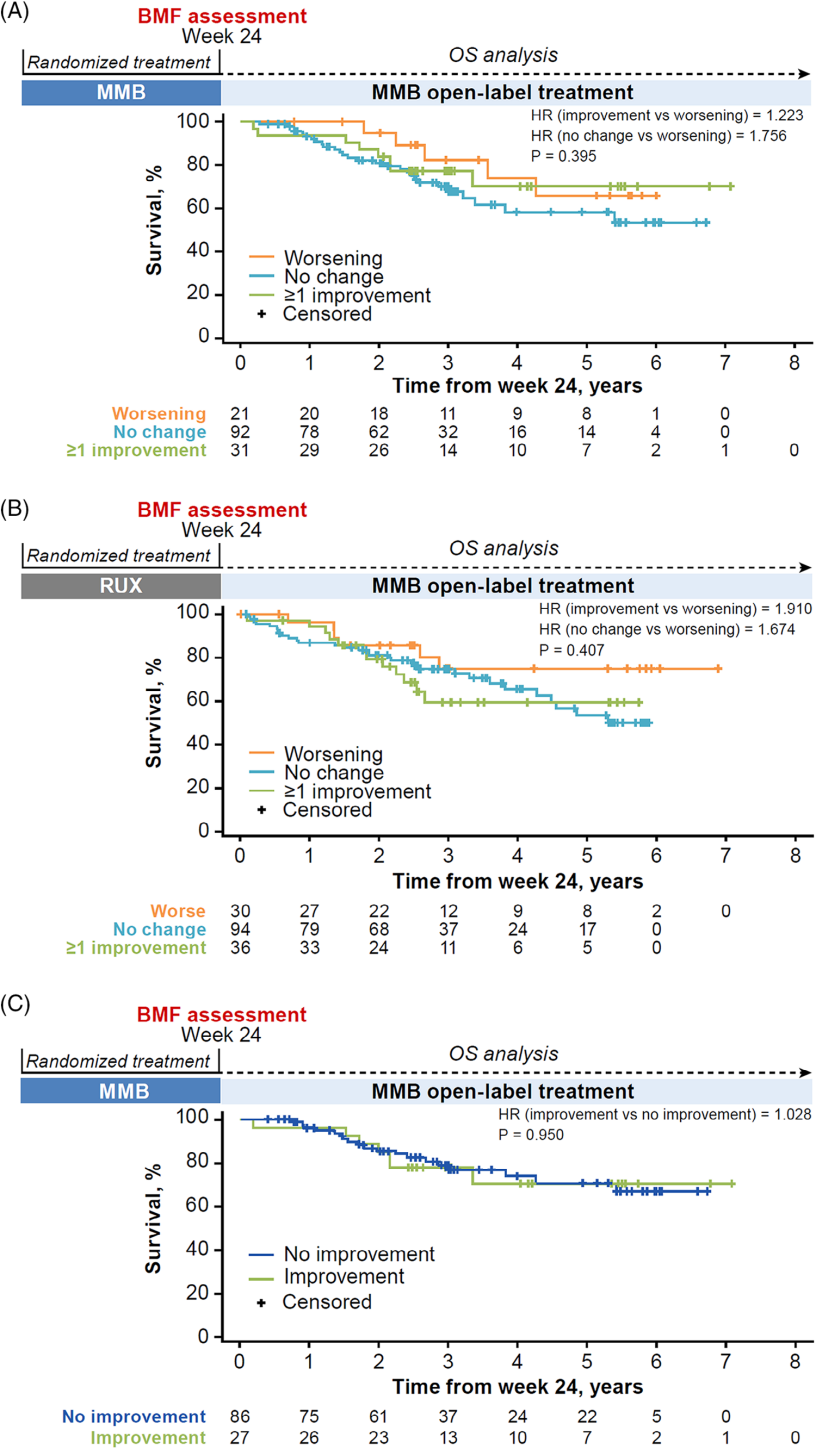
Overall survival from week 24 by baseline to week 24 BMF grade changes in JAK inhibitor–naive patients. (A) Patients in the momelotinib arm. (B) Patients in the ruxolitinib arm. (C) Patients in the momelotinib arm who achieved transfusion independence. BMF, bone marrow fibrosis; HR, hazard ratio; JAK, Janus kinase; MMB, momelotinib; OS, overall survival; RUX, ruxolitinib.

A landmark analysis of OS beginning at week 24, after patients in the ruxolitinib arm crossed over to receive open‐label momelotinib and patients in the momelotinib arm continued to open‐label momelotinib, is shown in Figure 7. The median follow‐up for OS was approximately 3 years. OS was analyzed by subgroups of change in BMF grades at week 24 within each treatment arm. During the momelotinib open‐label extended treatment period, changes in BMF grades at week 24 were not associated with OS benefit in either treatment arm. In the momelotinib‐randomized arm, OS rates at 2, 4, and 6 years were approximately 95%, 75%, and 65%, respectively, among patients with worsening BMF; 80%, 60%, and 55%, respectively, among patients with stable BMF; and 85%, 70%, and 70%, respectively, among patients with a ≥1‐grade improvement (Figure 7A). The Cox regression model showed no differences in OS in momelotinib‐randomized patients between those with a ≥1‐grade improvement vs worsening BMF (hazard ratio [HR], 1.22; 95% confidence interval [CI], 0.40–3.74; *p* = .725). In the ruxolitinib‐randomized arm, OS rates at 2, 4, and 6 years were approximately 85%, 75%, and 75%, respectively, among patients with worsening BMF; 80%, 65%, and 50%, respectively, among patients with stable BMF; and 80%, 60%, and 60%, respectively, among patients with a ≥1‐grade improvement (Figure 7B). The Cox regression model showed no differences in OS in ruxolitinib‐randomized patients between those with a ≥1‐grade improvement vs worsening BMF (HR, 1.91; 95% CI, 0.72–5.10; *p* = .196).

BMF grade improvements were also not associated with OS benefits in patients randomized to momelotinib who achieved transfusion independence at week 24 (HR [improvement vs no improvement], 1.03; *p =* .950) (Figure 7C).

## 4 DISCUSSION

Improvement in BMF has been pursued as an endpoint in clinical trials for myelofibrosis, especially in combination therapies, with a presumed link to disease modification [4, 36, 38]. Although studies have shown that the grade of BMF itself may directly influence OS [34, 37, 41], limited data, especially in the phase 3 setting, are available on how change or improvement in the grade of BMF potentially correlates with survival or other efficacy outcomes.

The current analysis included bone marrow biopsy samples from SIMPLIFY‐1, a large, completed phase 3 clinical trial of momelotinib vs ruxolitinib in JAK inhibitor–naive patients with myelofibrosis. The availability of such samples offered a unique opportunity to better understand the impact of two differentiated JAK inhibitors on BMF and the clinical consequences of changes in BMF. The proportions of each baseline BMF grade were similar between treatment arms, likely due to randomization stratification.

The data presented here showed modest improvement in BMF grade after 24 weeks of momelotinib or ruxolitinib treatment, with approximately 20% of patients having a ≥1‐grade improvement. In contrast, BMF was stable in > 60% of patients. In both arms, BMF grade change was not associated with symptom or spleen response. Although such similarities were observed, outcomes related to anemia were different between the two arms. For patients treated with momelotinib, hemoglobin levels increased, and transfusion independence was achieved regardless of BMF changes (improvement or worsening). In contrast, for patients treated with ruxolitinib, those with BMF improvement—even those achieving a 2‐grade BMF improvement—did not have improved hemoglobin levels, and worsening BMF grade was generally correlated with decreased hemoglobin levels. Changes in BMF grade also did not impact the achievement of transfusion independence in the ruxolitinib arm. These findings are suggestive of the previously described anemia benefit of momelotinib, a feature of its JAK1/JAK2/ACVR1‐mediated mechanism of action, which is not seen with ruxolitinib [42, 43]. Of note, not achieving transfusion independence was also not associated with changes in BMF grade.

Although SIMPLIFY‐1 was completed in 2019, the long‐term follow‐up of patients in the study and after rollover to the extended access protocol (NCT03441113) allowed OS assessment. The current analysis showed that patients achieving a ≥1‐grade BMF improvement did not demonstrate OS advantage in either of the treatment arms even with a longer follow‐up. Significant differences in OS were not evident among those with worsened, improved, or stable BMF for either arm.

A limitation of this analysis is that bone marrow biopsies were not centrally reviewed, which may have led to biases or inconsistencies in grading. Additionally, variation in bone marrow biopsies may lead to inadequate assessment of a patient's disease state; this is evident by the number of patients with grade 0 or 1 BMF with concurrent high clinical‐stage disease. Additionally, the analysis was performed at week 24, which may not have allowed for sufficient time to observe clinically relevant changes in BMF. Paired bone marrow biopsies were not available from all patients in the study; fewer biopsies were collected at week 24, indicating a potential skew in the sample pool. It is also notable that while the current analysis suggests that BMF may not necessarily be the appropriate surrogate of clinical response in the setting of first‐line JAK inhibitor monotherapy, the results cannot be generalized to myelofibrosis treatments with other mechanisms of action [39]. Furthermore, although the patient population was large, the number of patients having BMF improvement of ≥1 grade was low. Finally, although baseline genomic profiling may provide insights into potential biomarkers associated with BMF changes or other clinical endpoints, samples were not available at the time of this analysis. Nonetheless, this represents the most extensive analysis to date of the correlation of BMF changes with other outcome measures, with over 300 paired biopsies and mature clinical data, in JAK inhibitor–naive patients with myelofibrosis.

While the data continue to support the key role of ACVR1 inhibition together with JAK1 and JAK2 inhibition in the differentiated anemia benefit derived with momelotinib, they bring into question the use of BMF assessment at week 24 as a surrogate for clinical benefit and disease modification, given the lack of association between changes in BMF grade and OS and other efficacy outcomes. Emerging data from ruxolitinib and ruxolitinib combination therapies have shown approximately 30% of patients having a ≥1‐grade improvement in BMF; however, the improvement has not been significantly correlated with survival or other clinical outcomes [36, 37], except perhaps in individual cases [44, 45]. Future analyses of BMF grading incorporating additional bone marrow histopathology assessments such as megakaryocyte cell size and clustering, along with improved quantitative approaches for evaluating cytological and topographical bone marrow features, may be informative [46, 47].

## 5 CONCLUSION

In conclusion, the current analysis showed that both momelotinib and ruxolitinib can improve BMF grade, but the improvement was not associated with OS advantage or better efficacy outcomes; benefits of anemia with momelotinib were observed regardless of BMF changes. The clinical significance of BMF changes with nontransplant therapies for myelofibrosis should be carefully evaluated to better understand its role in disease modification, the pathogenesis of myelofibrosis, and clinically important outcomes.

## AUTHOR CONTRIBUTIONS

Jun Kawashima, Mei Huang, and Bryan Strouse contributed to the study design; Stephen T. Oh, Srdan Verstovsek, Vikas Gupta, Uwe Platzbecker, Timothy Devos, Jean‐Jacques Kiladjian, Donal P. McLornan, Andrew Perkins, Maria Laura Fox, Mary Frances McMullin, Adam J. Mead, Miklos Egyed, Jiri Mayer, Tomasz Sacha, Mei Huang, and Ruben Mesa contributed to data acquisition; Jun Kawashima, Mei Huang, and Bryan Strouse conducted the data analysis; Mei Huang performed the statistical analysis. All authors contributed to data interpretation, reviewed and provided important intellectual contributions to the manuscript, and approved the final version for publication.

## CONFLICT OF INTEREST STATEMENT

Stephen T. Oh reports consulting fees from AbbVie, Blueprint Medicines, Bristol Myers Squibb/Celgene, Constellation Pharmaceuticals, CTI BioPharma, Disc Medicine, Incyte, Kartos Therapeutics, PharmaEssentia, and Sierra Oncology. Srdan Verstovsek reports consulting fees from Bristol Myers Squibb/Celgene, Incyte, Novartis, and Sierra Oncology and research funding from AstraZeneca, Blueprint Medicines, Bristol Myers Squibb/Celgene, CTI BioPharma, Genentech, Gilead, Incyte, Italfarmaco, Novartis, NS Pharma, PharmaEssentia, and Promedior. Vikas Gupta reports consulting fees from AbbVie, Bristol Myers Squibb/Celgene, Constellation Biopharma, Novartis, Pfizer, and Sierra Oncology; honoraria from Bristol Myers Squibb/Celgene, Constellation Biopharma, and Novartis; and participation on a data safety monitoring board or advisory board for AbbVie, Bristol Myers Squibb/Celgene, Pfizer, and Roche. Uwe Platzbecker reports consulting fees from AbbVie, Bristol Myers Squibb/Celgene, Janssen, and Novartis; honoraria from Amgen, Jazz Pharmaceuticals, and Takeda; and participation on a data safety monitoring board or advisor board for AbbVie and Novartis. Timothy Devos reports consulting fees from AOP Health, Bristol Myers Squibb/Celgene, Incyte, and MorphoSys and honoraria from Novartis and Sobi. Jean‐Jacques Kiladjian reports honoraria from Novartis and participation on a data safety monitoring board or advisory board for AbbVie, AOP Orphan, Bristol Myers Squibb, Incyte, and Novartis. Donal P. McLornan reports grants or contracts from CPI and honoraria from AbbVie, Bristol Myers Squibb/Celgene, Jazz Pharmaceuticals, and Novartis. Andrew Perkins reports honoraria from AbbVie, Novartis, CTI BioPharma, Sierra Oncology, and Kartos Therapeutics. Maria Laura Fox reports consulting fees from AbbVie, GSK, Novartis, and Sierra Oncology; payment or honoraria from Bristol Myers Squibb and Novartis; and travel support from AbbVie. Mary Frances McMullin reports consulting fees from AbbVie, Bristol Myers Squibb, CTI, Novartis, and Sierra Oncology and honoraria from AbbVie, AOP, Incyte, Novartis, and Pfizer. Adam J. Mead reports consulting fees from AbbVie, Bristol Myers Squibb/Celgene, Galecto, Gilead, Incyte, Karyopharm, Novartis, Pfizer, Sensyn, and Sierra Oncology; travel fees from Bristol Myers Squibb/Celgene; and participation on a data safety monitoring board or advisory board for Bristol Myers Squibb/Celgene. Jiri Mayer reports research support from Sierra Oncology. Tomasz Sacha reports honoraria from Angelini Pharma, Bristol Myers Squibb/Celgene, Novartis, Pfizer, and Roche. Jun Kawashima reports employment at Sierra Oncology and stock or stock options at Gilead Sciences and Sierra Oncology. Mei Huang reports employment and stock options at Sierra Oncology. Bryan Strouse reports employment at Sierra Oncology. Ruben Mesa reports grants or contracts from AbbVie, Celgene, CTI Biopharma, Constellation Biopharma, Genotech, Incyte, Promedior, Samus Therapeutics, and the Mays Cancer Center P30 Cancer Center Support Grant from the National Cancer Institute (CA054174) and consulting fees from Constellation Biopharma, La Jolla, Novartis, and Sierra Oncology. Miklos Egyed declares no conflict of interest.

## FUNDING INFORMATION

This study was sponsored by Sierra Oncology, a GSK company.

## ETHICS STATEMENT

The study was approved by the institutional review boards and independent ethics committees at each study site, and all participants provided written informed consent. Additional signatures were collected for the use of biopsies in other biomarker analyses.

## CLINICAL TRIAL REGISTRATION

NCT01969838

## Supporting information



Supporting Information

## Data Availability

Sierra Oncology commits to sharing clinical study data with qualified researchers to enable the enhancement of public health. As such, Sierra will share anonymized patient‐level data on request or if required by law or regulation. Qualified scientific and medical researchers can request patient‐level data for studies of Sierra pharmaceutical substances listed on ClinicalTrials.gov and approved by health authorities in the USA and the EU. Patient‐level data for studies of newly approved pharmaceutical substances or indications can be requested 9 months after US Food and Drug Administration and European Medicines Agency approvals. Such requests are assessed at Sierra's discretion, and the decisions depend on the scientific merit of the proposed request, data availability, and the purpose of the proposal. If Sierra agrees to share clinical data for research purposes, the applicant is required to sign an agreement for data sharing before data release, to ensure that the patient data are de‐identified. In case of any risk of reidentification of anonymized data despite measures to protect patient confidentiality, the data will not be shared. The patient's informed consent will always be respected. If the anonymization process provides futile data, Sierra will have the right to refuse the request. Sierra will provide access to patient‐level clinical trial analysis datasets in a secured environment upon execution of the data‐sharing agreement. Sierra will also provide the protocol, statistical analysis plan, and the clinical study report synopsis if needed. For additional information or requests for access to Sierra clinical trial data for research purposes, please contact us at GSKClinicalSupportHD@gsk.com.
