# Correction to “Changes in bone marrow fibrosis during momelotinib or ruxolitinib therapy do not correlate with efficacy outcomes in patients with myelofibrosis”

**DOI:** 10.1002/jha2.934

**Published:** 2024-05-24

Oh S, Verstovsek S, Gupta V, et al. *eJHaem*. 2024;5(1):105‐116.

The authors wish to correct the following:

- In the Abstract, *p* values for spleen response were inadvertently indicated as referring to symptom response, and vice versa. Consistent with Figure 4, the text in question should have read “In addition, no associations between BMF changes and symptom (momelotinib, *p* = .126; ruxolitinib, *p* = .407)/spleen (momelotinib, *p* = .617; ruxolitinib, *p* = .833) outcomes were noted…”
- In paragraph 3 of the Introduction, the abbreviations for Mutation‐Enhanced International Prognostic Scoring System 70 and 70‐plus were incorrect and should have read “MIPSS70” and “MIPSS70+”
- In Figure 1, the label for the percentage of patients in the ruxolitinib arm with missing BMF grade was shown as 3%. As stated in the legend, 4/217 patients in that arm had missing data, so this label within the figure should have been 2%
- In Results Section 3.3, paragraph 1, the numbers of patients in the momelotinib arm with stable and worsening BMF who achieved a symptom response were inadvertently swapped. Consistent with the percentages stated and with Figure 4, the text in question should have read “…of the 89 patients (63%) with stable BMF, 27 (30%) achieved a symptom response; and of the 21 patients (15%) with worsening BMF, 11 (52%) had a symptom response…”

We apologize for these errors.

