# Supplementary material for: Changes in bone marrow fibrosis during momelotinib or ruxolitinib therapy do not correlate with efficacy outcomes in patients with myelofibrosis
Source: EJHaem. 2024 Feb 5;5(1):105–16. doi: 10.1002/jha2.854 (PMC10887367; doi:10.1002/jha2.854)
Supplement: Supplementary file 1 — Supporting Information [file JHA2-5-105-s001.docx]

**Figure S1.** **SIMPLIFY-1 study design and BMF analysis**.^1^ BMF, bone marrow fibrosis; JAKi, Janus kinase inhibitor; OS, overall survival; RT, randomized treatment.


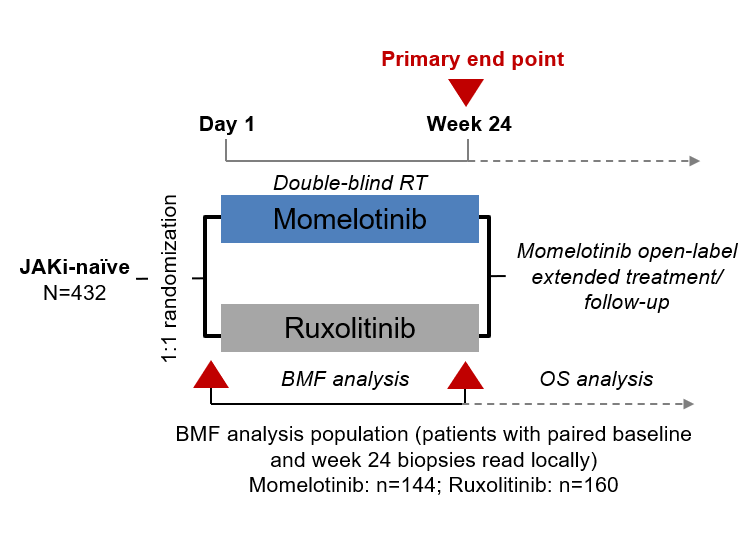


**Figure S2.** **Hemoglobin levels at baseline and week 24 in JAK inhibitor–naive patients in SIMPLIFY-1 with improved BMF grade.** (A) Patients treated with momelotinib. (B) Patients treated with ruxolitinib. BMF, bone marrow fibrosis; JAK, Janus kinase.


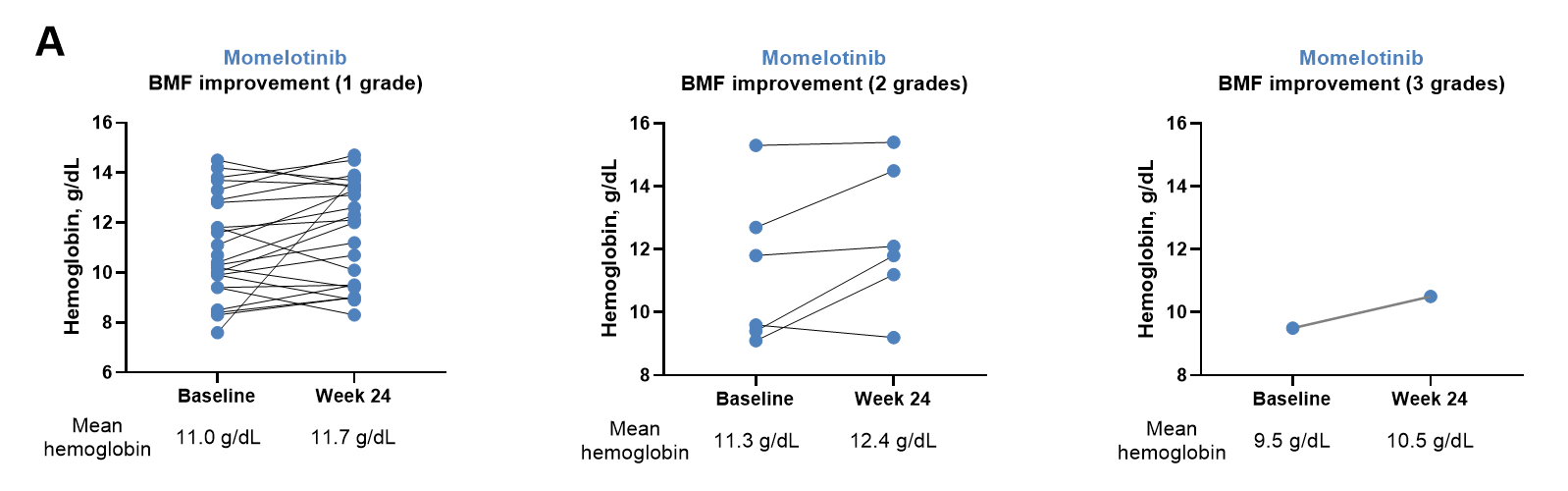


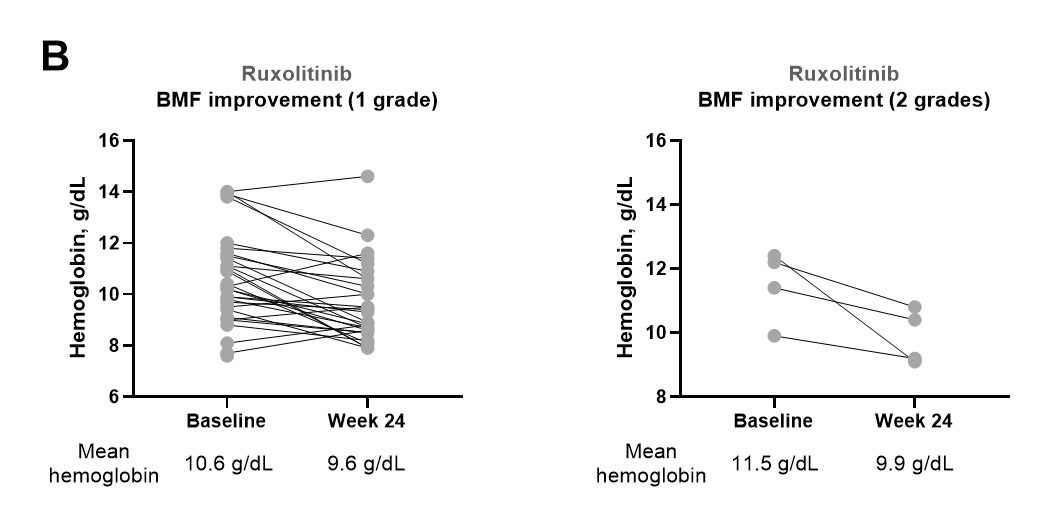


**Supplementary Reference**

1. Mesa RA, Kiladjian JJ, Catalano JV, Devos T, Egyed M, Hellmann A, et al. SIMPLIFY-1: A phase III randomized trial of momelotinib versus ruxolitinib in Janus kinase inhibitor-naive patients with myelofibrosis. J Clin Oncol. 2017;35(34):3844-50.
